# Cationic Nanocylinders Promote Angiogenic Activities of Endothelial Cells

**DOI:** 10.3390/polym8010015

**Published:** 2016-01-14

**Authors:** Jung Bok Lee, Daniel A. Balikov, Jae Won Yang, Ki Seok Kim, Hun Kuk Park, Jeong Koo Kim, Il Keun Kwon, Leon M. Bellan, Hak-Joon Sung

**Affiliations:** 1 Department of Biomedical Engineering, Vanderbilt University, Nashville, TN 37212, USA; jung.bok.lee@vanderbilt.edu (J.B.L.); daniel.a.balikov@vanderbilt.edu (D.A.B.); jkkim@inje.ac.kr (J.K.K.); leon.bellan@vanderbilt.edu (L.M.B.); 2 Department of Mechanical Engineering, Vanderbilt University, Nashville, TN 37235, USA; 3 Department of Internal Medicine, Yonsei University of Wonju College of Medicine, Wonju, Gangwon 220-701, Korea; kidney74@yonsei.ac.kr; 4 Department of Medical Engineering, College of Medicine, Kyung Hee University, Seoul 130-701, Korea; inwoodkr@gmail.com (K.S.L.); sigmoidus@khu.ac.kr (H.K.P.); 5 Department of Biomedical Engineering, Inje University, Gimhae 621-749, Korea; 6 Department of Maxillofacial Biomedical Engineering and Institute of Oral Biology, School of Dentistry, Kyung Hee University, Seoul 130-701, Korea; kwoni@khu.ac.kr; 7 Division of Cardiovascular Medicine, Vanderbilt University, Nashville, TN 37235, USA; 8 Severance Biomedical Science Institute, Yonsei University College of Medicine, 50 Yonsei-ro, Seodaemun-gu, Seoul 120-749, Korea

**Keywords:** cationic nanocylinder, cell–cell interaction, endothelial cells, migration, tubulogenesis

## Abstract

Polymers have been used extensively taking forms as scaffolds, patterned surface and nanoparticle for regenerative medicine applications. Angiogenesis is an essential process for successful tissue regeneration, and endothelial cell–cell interaction plays a pivotal role in regulating their tight junction formation, a hallmark of angiogenesis. Though continuous progress has been made, strategies to promote angiogenesis still rely on small molecule delivery or nuanced scaffold fabrication. As such, the recent paradigm shift from top-down to bottom-up approaches in tissue engineering necessitates development of polymer-based modular engineering tools to control angiogenesis. Here, we developed cationic nanocylinders (NCs) as inducers of cell–cell interaction and investigated their effect on angiogenic activities of human umbilical vein endothelial cells (HUVECs) *in vitro*. Electrospun poly (l-lactic acid) (PLLA) fibers were aminolyzed to generate positively charged NCs. The aninolyzation time was changed to produce two different aspect ratios of NCs. When HUVECs were treated with NCs, the electrostatic interaction of cationic NCs with negatively charged plasma membranes promoted migration, permeability and tubulogenesis of HUVECs compared to no treatment. This effect was more profound when the higher aspect ratio NC was used. The results indicate these NCs can be used as a new tool for the bottom-up approach to promote angiogenesis.

## 1. Introduction

Polymers have been extensively used as one of the major materials for tissue engineering and regeneration over the past several decades due to their tunable properties [1,2]. In particular, modification of polymeric scaffolds, surfaces and nanoparticles have been studied to promote microvasculature formation within damaged or engineered tissues [1,3]. Angiogenesis, formation of new blood vessels from existing ones, is required to provide oxygen and nutrient to cells within these tissues. However, promoting angiogenesis to sufficiently support survival, growth and function of cells within the tissues is still considered challenging due to spatiotemporal variations in pro-angiogenic effects generated by small molecule delivery as well as scaffold or surface fabrication [4,5]. The emergence of modular tissue engineering resulted in a paradigm shift from top-down to bottom-up approaches in instructing cellular and tissue responses [6]. In bottom-up approaches, micro- or even nanoscale building blocks can be assembled to provide higher resolution guidance on complex tissue morphogenesis (e.g., sheet-based tissue modules, cell-laden hydrogels and tissue printing) [7,8,9]. This strategy can be applied to mitigate spatiotemporal variations of pro-angiogenic activities resulting from the current approaches.

Endothelial cells (ECs) organize as a monolayer on the luminal face of blood vessels and contribute to vascular homeostasis in both large and small vessels. Angiogenesis involves migration, elongation and self-assembly of ECs as the arteriole and capillary scales [10,11]. Robust cell–cell interaction of ECs is required to promote their assembly and subsequent tube formation, sprouting and connection with neighboring vessels that comprise the vascular networks, thereby supporting larger tissue masses. Endothelial junctions among ECs play pivotal roles in maintaining vascular permeability, tissue integrity, barrier function and cell–cell communication during angiogenesis [12]. Compared to delivery of pro-angiogenic molecules via nanoparticles or scaffolds, physically improving cell–cell interaction of ECs through a bottom-up approach is more advantageous in promoting angiogenesis since manipulating downstream cellular processes of angiogenesis is comparatively easier than altering upstream molecular mechanisms. In this way, spatiotemporal variations of pro-angiogenic activities can be mitigated, and biological side effects on alteration of other cell behaviors can be minimized [11]. Progress has been made in developing scaffold-free cell-assembled devices using technologies such as magnetic nanoparticles and fibrin microbeads that better instruct ECs during angiogenesis and even contribute to neotissue formation [13,14].

As a part of the aforementioned ongoing efforts, we developed cationic nanocylinders (NCs) as inducers of cell–cell interaction and investigated their effect on angiogenic activities of human umbilical vein endothelial cells (HUVECs) *in vitro*. Electrospun poly (l-lactic acid) (PLLA) fibers with a mean diameter of 290 ± 50 nm were aminolyzed to generate positively charged NCs. The aninolyzation time was changed to produce two different aspect ratios (*i.e.*, 7 and 40) of NCs. When HUVECs were treated with NCs, the electrostatic interaction of cationic NCs with negatively charged plasma membranes promoted HUVEC migration, permeability and tubulogenesis compared to no treatment. This effect was more profound when the longer NC (higher aspect ratio: 40) was used, compared to the shorter NC (lower aspect ratio: 7). These results indicate the NCs can be used as a new tool for the bottom-up approach to promote angiogenesis and consequent tissue regeneration.

## 2. Experimental Section

### 2.1. Fabrication of PLLA Nanocylinders (NCs)

Poly (l-lactic acid) (PLLA, RESOMER^®^ L207 S, i.v. = 1.5–2.0 dL/g, Boehringer Ingelheim Pharma GmbH & Co. Fine Chemical, Ingelheim am Rhein, Germany) was dissolved in 1,1,1,3,3,3-hexafluoro-2-propanol (HFIP) at a concentration of 4 wt %. The PLLA/HFIP solution was electrospun through a 22-gauge needle at a 1 mL/h feed rate under 18 kV voltage with a distance of 10 cm between the needle tip and rotating collector (10 cm diameter). The relative humidity in the electrospinning chamber was held at 20% during electrospinning using a humidity controller (5200, Electro-tech Systems, Hammond, IN, USA). To produce two different aspect ratios of PLLA NCs, aspect ratio small (ARS) and aspect ratio large (ARL), electrospun PLLA nanofiber mats were aminolyzed by immersing in 10 wt % of 1,6-hexanmethylenendiamine (HMDA, Sigma-Aldrich, St Louis, MO, USA) isopropanol solution, and allowed to react at 37 °C over different time periods (60 and 90 min), respectively. Aminolyzed products were washed three times with cold ethanol. To introduce the fragmentation for NCs, ultra-sonication was performed on the aminolyzed nanofibers in 50% ethanol at 4 °C for 30 min. The resulting PLLA NCs were lyophilized and resuspended in PBS at desired concentrations. To visualize NCs by confocal microscopy, NCs were tagged with Alexa Fluor 647 NHS Ester (Molecular Probes, ThermoFisher Scientific, Waltham, MA, USA). 20 mg of NCs was suspended in 1 mL of 0.1 mM sodium bicarbonate buffer and mixed with reactive fluorophore solution (10 μL of 0.1μg/μL). The suspension was allowed to react for 3 h with occassional vortexing. Tagged NCs were then washed with PBS and stored at −80 °C after lyophilization.

### 2.2. Characterization of PLLA NCs

ARS and ARL NCs were imaged using a scanning electron microscope (SEM, Hitachi S 4200, Tokyo, Japan) after sputter coating with gold for 1 min and their diameter and aspect ratio were analyzed. To verify the presence of amine groups (NH_2_) on the NC surface, which encourages electrostatic interaction with cell membrane, Fluorescamine (Sigma-Aldrich, St. Louis, MO, USA) was treated. Briefly, 1 mg of NCs were immersed in 3 mg/mL Fluorescamine/PBS solution and allowed to react with primary amines on the NCs at room temperature for 15 min. Fluorescence measurements were detected at an emission wavelength of approximately 470 nm using a fluorospectrometer (UV-1650PC, Shimadzu, Kyoto, Japan). The average weight molar mass (*M*_w_ and *M*_n_) and polydispersity index (PDI) of aminolyzed PLLA NCs were measured by gel permeation chromatography (GPC).

### 2.3. Cell Culture

Red fluorescent protein- human umbilical vein endothelial cells (RFP-HUVECs, passages 6–8) were cultured on 0.1% gelatin coated T-175 cell culture flasks with Endothelial cell Growth Medium (EGM-2, Lonza, Atlanta, GA, USA) containing human epidermal growth factor (EGF), hydrocortison, GA-1000 (Gentamicin, Amphotericin-B), 2% Fetal Bovine Serum (FBS), vascular endothelial growth factor (VEGF), human fibroblast growth factor (hFGF)-B, R3-IGF-1, ascorbic acid, heparin, and 1% penicillin/streptomycin.

### 2.4. HUVEC Migration Assay

A scratch wound migration assay was conducted to examine the effect of NCs on RFP-HUVEC migration. HUVECs were seeded onto 24-well plates (20,000/well) with or without 0.l% gelatin coating. After a confluent monolayer was formed, a straight line of wound area was created by scratching the well surface with a 200 μL pipet tip, followed by treatment of ARS or ARL NCs at 50 ug/mL. Cell migration was observed at 0, 6 and 12 h post scratch to evaluate closure of the wound area by confocal microscopy (LSM710, Carl Zeiss, Oberkochen, Germany)[15]. The images acquired for each sample were analyzed using ImageJ by measuring the gap distance between the scratch edges.

### 2.5. Permeability Test

HUVECs were seeded with a density of 20,000/well on 0.4 μm pore polycarbonate transwell filters (Corning Coster, Cambridge, MA, USA) and cultured for 2 days to achieve confluent monolayers. FITC-dextran microparticles (1 mg/mL, *M*_w_ = 10,000, Sigma-Aldrich, St. Louis, MO, USA) were added onto transwell filters and allowed to penetrate through the cell monolayers. At 1, 3, 6, and 24 h, 100 μL of culture media containing penetrated microparticles was taken from the lower compartment of the transwell. The fluorescence signal of the flow-through media was quantified with a microplate reader (Infinite^®^ M1000 pro, TECAN, Zurich, Switzerland) at excitation/emission wavelengths of 492/520 nm [16].

### 2.6. In Vitro Tubulogenesis

Tubulogenesis of HUVECs in response to NC treatment was examined on growth factor reduced-Matrigel (Corning, NY, USA) diluted 1:1 with serum-free DMEM [17]. Briefly, HUVECs with a density of 10,000 cells/cm^2^ were seeded on Matrigel-coated 24-wells with or without treatment of NCs at 50 μg/mL. Tube formation of HUVECs was observed at 6, 12, and 24 h using confocal microsocpy, and total tube length, loop number, and branching points were analyzed using the Angiogenesis Analyzer plugin of ImageJ (Gilles Carpentier, Faculté des Sciences et Technologie, Université Paris Est, Creteil Val de Marne, France).

### 2.7. Quantitative Reverse Transcriptase-Polymerase Chain Reaction (qRT-PCR)

After 12 h on growth factor reduced Matrigel, with or without NCs, HUVECs were homogenized with Trizol reagent (Life Technologies, Carlsbad, CA, USA), mixed with chloroform (1:5 Trizol:chloroform, Sigma Aldrich, St. Louis, MO, USA), and separated by centrifugation (12,000 g, 15 min, 4 °C). The aqueous phase containing RNA was isolated using RNeasy columns (Bio-Rad, Hercules, CA, USA) according to the manufacturer’s instructions. RNA concentration was determined using a TECAN M1000 plate reader with manufacturer’s software. cDNA was synthesized using a cDNA generation kit (Applied Biosystems, Life Technologies, Carlsbad, CA, USA) and qPCR was performed using the SYBR Green master mix kit (Bio-Rad, Hercules, CA, USA) with 15 ng cDNA and 500 mM each of forward and reverse primers (Supplementary Materials Table S1). A CFX Real-Time PCR System (Bio-Rad, Hercules, CA, USA) ran the qPCR reaction. Expression of each gene was normalized to the expression of glyceraldehyde 3-phosphate dehydrogenase (GAPDH) as a housekeeping gene, thereby generating Δ*C*(t) values, and expression of 2^−ΔΔ*C*(*t*)^ relative to the TCPS control (*N* = 3 biological replicates for each experiment) [18].

### 2.8. Statistical Analysis

All quantitative data was presented as one standard deviation of the mean. One-way ANOVA was used to calculate the difference among groups. A *p*-value less than 0.05 was considered significant.

## 3. Results and Discussion

### 3.1. NC Characterization

**Figure 1:**
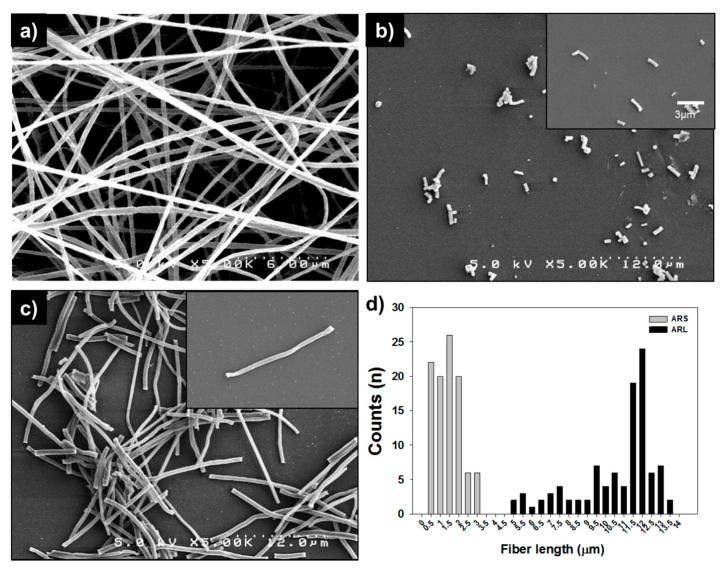
Scanning electron microscope (SEM) images of (**a**) electrospun PLA nanofibers before and (**b**,**c**) after aminolyzation to different aspect ratios nanocylinders; (**b**) aspect ratio small (ARS) and (**c**) aspect ratio large (ARL); and (**d**) distribution of NC length.

PLLA nanofibers with a mean diameter of 290 ± 50 nm were produced by electrospinning (Figure 1a). As has been previously reported, when semi-crystalline polymers such as PLLA undergo aminolysis, the amorphous region of PLLA degrades preferentially leaving crystalline regions of PLLA [19,20]. Additionally, crystallized PLLA nanofibers are easily broken down in response to shear-stress [21,22,23]. During this amine reactive degradation process, the diamine attacks ester groups of the PLLA backbone, producing amine (NH_2_) groups on the PLLA NCs [24,25]. As we found previously, COO– and C–O–C of the PLLA backbone were reacted with diamines by aminolysis, resulting in production of amine groups on the NC surface [26]. This amine-based cationic surface of NCs mediates electrostatic interactions with the negatively charged cell membrane, thereby promoting cell–cell interaction. Using the aforementioned amine reactive degradation process (*i.e.*, alkaline catalyzed degradation), the PLLA nanofibers were converted into cylindrical fragments while under ultra-sonication. The amine content of NC-ARS and NC-ARL were 7.05 ± 2.24 and 6.02 ± 1.23 μmol/g, respectively. The molecular weight of electrospun PLLA nanofiber was initially 200,000 g/mol and became 50,000 g/mol after 30 min and 23,000 g/mol after 1 h aminolysis reactions. SEM images demonstrate that NCs were successfully produced utilizing two different aminolysis reaction times (60 and 90 min) generating NCs with an aspect ratio of 7 (ARS) and 40 (ARL) (Figure 1b,c). Quantitative analysis of SEM images confirmed that the distribution of NC length between ARS and ARL did not overlap with each other (Figure 1d).

### 3.2. Cell Migration Study

For all biological studies, NCs were cultured with red fluorescent protein–human umbilical vein cells (HUVECs) as the model cell type to explore the NC’s effect on angiogenic behavior of HUVddddECs. When compared with untreated HUVECs, there was no observed cytotoxicity caused by the NCs in the concentration range of 1–100 μg/mL (data not shown). Of the many assays designed to test endothelial cell function, the *in vitro* scratch wound assay is chosen because the assay procedure is simple, and it allows for investigation of cell- matrix and cell–cell interactions during the migration process. In other popular methods, such as Boyden chamber assays, preparation of cells in suspension before the assays disrupts cell–cell and cell–ECM interactions, thereby biasing the cells out of the monolayer setting [15]. Overall, the scratch wound healing assay is an economical and easy method to study cell migration because the cells migrated into the newly created gap (“scratch”) and closed the gap as new cell–cell contacts are established yielding a simple assay readout [15,27].

**Figure 2:**
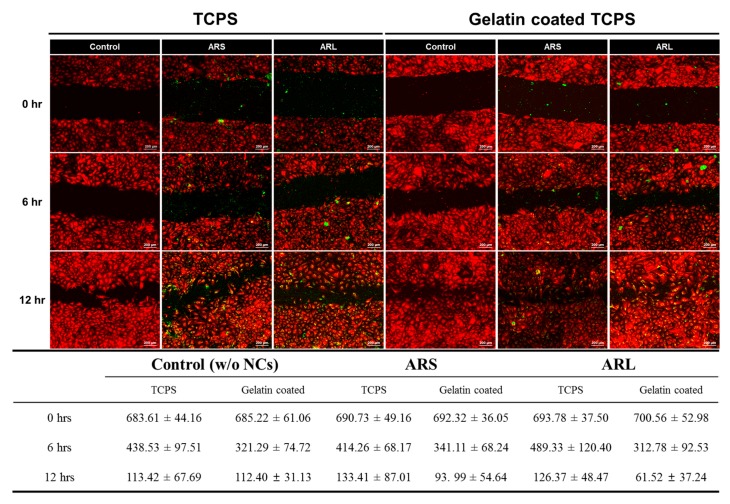
Analysis of HUVECs migration by *in vitro* scratch assay. Images were acquired at 0, 6, and 12 h post scratch generation. The degree of migration was determined by quantifying the number of cells migrated to the center of scratch. (Red: RFP HUVECs; Green: Alexa Fluor 647 tagged NCs).

In order to study HUVEC migration activity, we carried out a wound scratch assay on both TCPS and 0.1% gelatin coated TCPS surfaces over a 12 h period to account for different substrates that have been used as test surfaces in the literature (Figure 2). Upon initially creating the scratch at 0 h, all the gaps were between 680 and 700 μm. For both substrates, the HUVECs migrated into the scratch wound, but the average wound gap after 12 h was smaller across all test conditions on the gelatin-coated surface compared to TCPS (Gelatin control: 112.40 ± 31.13 μm, Gelatin ARS: 93.99 ± 54.64 μm, Gelatin ARL: 61.52 ± 37.24 μm). Interestingly, the average gap size was larger than the untreated control condition for both NC sizes on the TCPS substrate (TCPS control: 113.42 ± 67.69 μm, TCPS ARS: 133.41 ± 87.01 μm, TCPS ARL: 126.37 ± 48.47 μm). Considering the fact that gelatin serves as a standard coating material for endothelial cell culture, HUVEC migration might be facilitated to the degrees seen in a physiological condition by gelatin-mediated repeated formation and destruction of focal adhesion while migration. However, this facilitation function was not sufficiently active in TCPS. Between the two NC aspect ratios, the ARL condition induced more migration of HUVECs compared to the ARS condition likely due to some form of contact guidance provided by NCs with the larger aspect ratio. This feature of ARL likely provides a fibrillar structure similar to that of basement membrane, which the HUVECs are accustomed to growing on. Recognition of this geometrical motif may explain the better migration performance of ARL over ARS. ARS has more of a particle geometry, which is more foreign to stromal structure on the abluminal surfaces of blood vessels compared to luminal free-floating particles in the blood stream itself. Particles are more likely engulfed through endocytosis mechanisms and ARS may be recapitulating this phenomenon within this experimental setting.

### 3.3. Permeability Test

**Figure 3:**
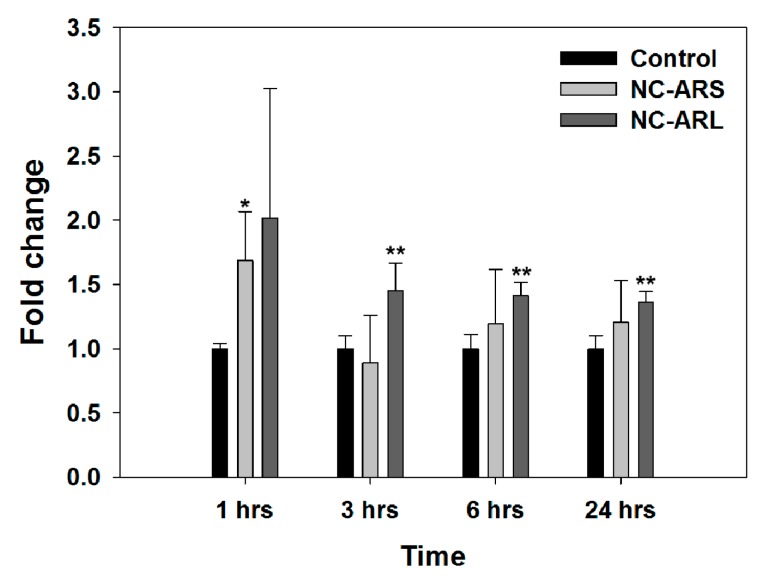
NCs induce endothelial cell permeability *in vitro*. Quantitative transwell assay to measure HUVEC permeability showed increased permeability after treating with NCs. (* *p* < 0.05, ** *p* < 0.01 relative to control).

The effect of NCs on HUVEC monolayer integrity was investigated. To quantify permeability, a confluent monolayer of HUVECs was plated over 0.4 μm porous Transwell insert. FITC-dextran microparticles were then added to the upper chamber. The microparticles penetrated through the HUVEC monolayer were collected in the bottom chamber, and their fluorescence intensity was measured. The permeability of dextran microparticles increased when HUVECs were treated with NCs (Figure 3). After 1 h, the ARS group had a significant increase in FITC signal compared to the control condition. However, at the 3, 6 and 24 h time points, the ARL group had significant higher FITC signal relative to the control condition. Given these data, the ARS group likely caused initial cell aggregation in local areas where ARS NCs are placed. However, from 3 h post treatment, the HUVECs could compensate against the ARS NCs, while the ARL NCs began to induce local cell aggregation and thus increase permeability in the cell monolayer. These results indicate NC treatment disrupts uniform cell–cell junction formation of HUVECs over the monolayer culture area but promotes local cell–cell aggregation that is usually required to facilitate tubulogenesis and sprouting processes of ECs undergoing angiogenesis. NC treatment may not be an ideal method to improve formation of cell–cell junction in a uniform monolayer fashion but an excellent tool to induce local EC assembly towards tubulogenesis.

### 3.4. Tubulogenesis In Vitro

**Figure 4:**
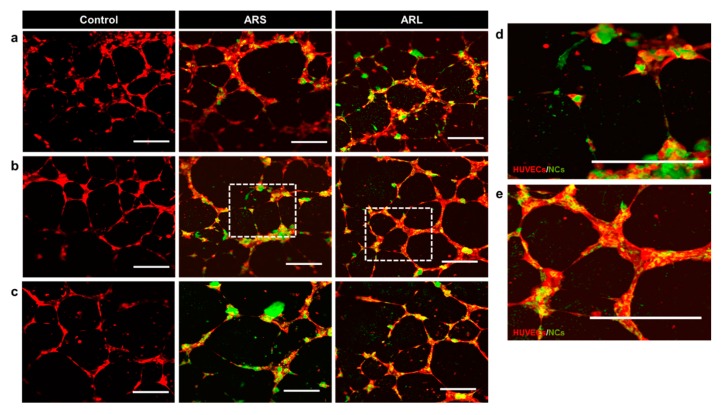
Confocal images of human umbilical vein cells (HUVECs) cultured on matrigel substrates when NCs were treated for (**a**) 6 h; (**b**) 12 h; and (**c**) 24 h (red: RFP-HUVECs/green: Alexa 647-tagged NCs). Zoomed images of HUVECs cultured with (**d**) ARS and (**e**) ARL after 12 h. Scale bar = 200 μm.

Because the results from the migration and permeability assays indicate that NC treatment may promote angiogenic activities of endothelial cells as evidenced by increased migration and local cell assembly, tubulogenesis of HUVECs was determined with or without NC treatment. During angiogenesis, local cell–cell interaction plays a central role in successful vessel formation and stabilization [28]. HUVECs were cultured on top of growth factor reduced Matrigel and treated with NCs. NCs localized in any particular tube structure were visualized to gain insight into a mechanism that NCs exploit in affecting tubulogenic behavior of HUVECs. Over 24 h, the HUVECs displayed very different structural morphologies across the tested culture conditions (Figure 4). At 6 h, the ARS group showed distinct larger clustering of NCs (shown in green) while the general tube structures appeared wider than either control or ARL groups. In the ARL group, the NCs formed some larger aggregates; however, small green punctate staining within the tubes of the empty Matrigel surface demonstrates a range of NC clustering. Similar to ARS, the tube diameter appeared larger than the control group.

At 12 h, the tubular structures began to change. For ARS, the NC aggregates became larger and inhibited continuous tube formation or stabilization as shown in Figure 4d. The NCs form HUVEC nodules at the branching points as the tubes begin to recede. On the other hand, the ARL condition shows the NC aggregates becoming smaller and in fact elongating along the long axis of the HUVEC tubes (Figure 4e) while creating larger diameter tubes compared to either control or ARS group. Finally, at 24 h, the control and ARS group show the degradation of tube structures while the ARL group maintained the highly distributed NCs along the HUVEC tubes, which in this assay normally are not sustained beyond 18 h.

**Figure 5:**
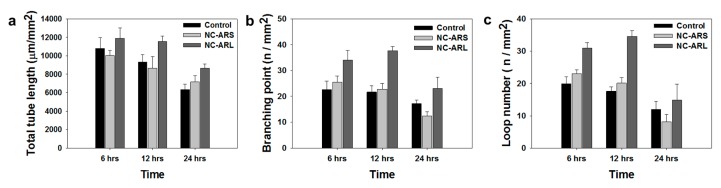
The effect of NCs on capillary-like structure formation: (**a**) total tube length; (**b**) branching points; and (**c**) loop numbers of capillary-like structures of HUVECs on Matrigel without (control) or with NC treatment.

Analyzing all the images quantitatively (Figure 5), the ARL condition consistently had the highest total tube length, number of branching points and loop number among all tested conditions. It is evident from the literature that many variables impact nanoparticles uptake into cells including size, shape, and surface charge [12,13,14,15]. Moreover, it is well known that positively charged nanoparticles below a certain size threshold are well-suited for endocytic processing by cells as they can interact favorably with the negatively charged phospholipid components of the cell membrane [12]. As a result, the impact of surface chemistry on cell–nanoparticle (or in this study’s case—nanomaterials) interaction and cellular uptake is being recognized and heavily considered in current and future research [16,17]. Overall, a total tube length was decreased by the passage of time. And branching point and loop number were decreased at 24 h. The *in vitro* formation of capillary-like structures by endothelial cells on the matrigel is a rapid, quantitative and reliable *in vitro* angiogenesis assay. When the endothelial cells are plated on Matrigel, they formed tubes within 6 h. When they formed tube structures, they start to detach from the matrix and break apart (around 12 h) and finally undergo apoptosis after 24 h. We analyzed this angiogenesis assay with three time periods that can be evidence of roll of NCs on the stability of tube formation. Capillary-like structures by HUVECs with ARL showed highest level of each factors and breaking ratio of tube structure was more delayed than the other groups on 12 and 24 h.

Hence, for this study, the results imply that the NCs had a beneficial effect on angiogenesis over the long term when they have larger aspect ratios, while they prove not very efficient with smaller aspect ratios in the *in vitro* setting. The results suggest the NCs as a new tool to continue studying bottom-up modulation of HUVECs for sustained tubulogenesis and perhaps a means to investigate a signaling cascade related angiogenic behavior.

### 3.5. Gene Expression

**Figure 6:**
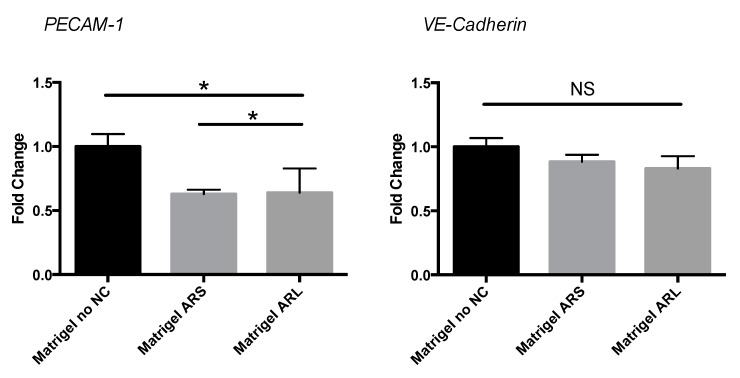
Quantitative real time PCR of angiogenic gene expression in HUVECs on Matrigel with NCs. Results are expressed as mean values ± S.D. * statistical significance at the level of *p* < 0.05.

Because the *in vitro* angiogenesis results differed dramatically among the tested groups, we desired to see what angiogenic cell–cell adhesion genes were being modulated during the angiogenesis assay. RNA was harvested 12 h after seeding the same angiogenesis assay as described in Section 3.4 and processed for quantitative PCR (Figure 6). Platelet endothelial cell adhesion molecule 1 (PECAM-1) did show significant differences in expression levels compared to the control group with decreased expression for both ARS and ARL, whereas vascular endothelial cadherin (VE-Cadherin) did not show significant differences. Both genes are known to be directly involved in angiogenesis and cell–cell junctions for endothelial cells [16]. Therefore, the one-sided response in *PECAM-1* over *VE-Cahderin* may indicate a preferential interaction that the NCs replace or compliment; hence the need for *PECAM-1* could be decreased.

## 4. Conclusions

Endothelial cell–cell interaction is required to promote tube formation, sprouting and interconnection as interendothelial junction formation plays pivotal roles in assembly and communication of endothelia cells. As a part of ongoing efforts to establish bottom-up approaches in instructing cell and tissue responses, cationic nanocylinders with two different aspect ratios were developed by applying electrospinning and aminolyzation techniques. These nanocylinders induced endothelial cell–cell interaction through electrostatic interactions with negatively charged cell membranes and thereby promoted migration, permeability, and tubulogenesis of endothelial cells compared to no treatment. This effect was more profound when nanocylinders with the higher aspect ratio were treated to cells. These results suggest NCs as a new modular tissue engineering tool to provide a high resolution instruction on cell–cell interaction and subsequent tubulogenic response of endothelial cells.

## Supplementary Materials

Supplementary materials can be found at www.mdpi.com/2073-4360/8/1/15/s1.
