# Supplementary material for: Cationic Nanocylinders Promote Angiogenic Activities of Endothelial Cells
Source: Polymers (Basel). 2016 Jan 14;8(1):15. doi: 10.3390/polym8010015 (PMC5639919; doi:10.3390/polym8010015)
Supplement: Supplementary file 1 [file polymers-08-00015-s001.pdf]

Polymers **2016**, *8*, 15

Supplementary Materials: Cationic Nanocylinders Promote Angiogenic Activities of Endothelial Cells

Jung Bok Lee, Daniel A. Balikov, Jae Won Yang, Ki Seok Kim, Hun Kuk Park, Jeong Koo Kim, Il Keun Kwon, Leon M. Bellan and Hak-Joon Sung

Table S1. Primer sequences for qPCR.

Gene name	Accession #	Forward primer 5′->3′	Reverse primer 5′->3′	Amplicon length (bp)
PECAM-1	NM_000442.4	CCAAGCCCGAACTGGAATCT	CACTGTCCGACTTTGAGGCT	168
VE_Cadherin	NM_001795.3	GCCAGTTCTTCCGAGTCACA	TTTCCTGTGGGGGTTCCAGT	130
Angiopoeitin 1	NM_001146.3	GCTGACAGATGTTGAGACCCA	TTCCTCCCTTTAGTAAAACACCTTC	395
Angiopoeitin 2	NM_001147.2	AACTGATGTGGAAGCCCAAGT	GGGTCCTTAGCTGAGTTTGATGT	387
Tie 1	NM_005424.4	TCTCATGTGGGCGCGG	AGCGGTGTCACCTTTGTTCA	378
Tie 2	NM_000459.3	GGATAGGGCTTGAGTGCCC	ACCTTCCACAGTTCCAGAAAGG	356
GAPDH	NM_002046.4	GCACCGTCAAGGCTGAGAAC	TGGTGAAGACGCCAGTGGA	138

© 2016 by the authors; licensee MDPI, Basel, Switzerland. This article is an open access article distributed under the terms and conditions of the Creative Commons by Attribution (CC-BY) license (http://creativecommons.org/licenses/by/4.0/).
